# The Spontaneous Course of Human Herpesvirus 6 DNA-Associated Myocarditis and the Effect of Immunosuppressive Intervention

**DOI:** 10.3390/v14020299

**Published:** 2022-01-31

**Authors:** Ahmed Elsanhoury, Uwe Kühl, Bruno Stautner, Oliver Klein, Alexander Krannich, Daniel Morris, Monika Willner, Ewa Jankowska, Karin Klingel, Sophie Van Linthout, Carsten Tschöpe

**Affiliations:** 1 Berlin Institute of Health at Charite (BIH)-Universitätmedizin Berlin-BIH Center for Regenerative Therapies (BCRT), 13353 Berlin, Germany; ahmed.elsanhoury@charite.de (A.E.); uwe.kuehl@charite.de (U.K.); brunostautner@googlemail.com (B.S.); oliver.klein@charite.de (O.K.); sophie.van-linthout@charite.de (S.V.L.); 2 German Centre for Cardiovascular Research (DZHK), Partner Site Berlin, 13353 Berlin, Germany; 3 Department Internal Medicine and Cardiology, Charité—University Medicine Berlin, Campus Virchow Klinikum, 13353 Berlin, Germany; daniel-armando.morris@charite.de (D.M.); monika.willner@charite.de (M.W.); 4 Institute of Heart Diseases, University Hospital Wroclaw, 50-556 Wroclaw, Poland; ewa.jankowska@umw.edu.pl; 5 Experimental and Clinical Research Center (ECRC), Charité—Universitätsmedizin Berlin, 13125 Berlin, Germany; alexander.krannich@charite.de; 6 Institute of Heart Diseases, Wroclaw Medical University, 50-367 Wroclaw, Poland; 7 Cardiopathology, Institute for Pathology and Neuropathology, University Hospital Tübingen, 72076 Tübingen, Germany; Karin.Klingel@med.uni-tuebingen.de

**Keywords:** myocarditis, human herpesvirus 6, immunosuppression

## Abstract

Introduction: This study investigated the spontaneous clinical course of patients with endomyocardial biopsy (EMB)-proven lymphocytic myocarditis and cardiac human herpesvirus 6 (HHV6) DNA presence, and the effectiveness of steroid-based intervention in HHV6-positive patients. Results: 756 heart failure (HF) patients underwent an EMB procedure to determine the underlying cause of unexplained HF. Low levels of HHV6 DNA, detectable by nested PCR only, were found in 10.4% of the cases (*n* = 79) of which 62% (*n* = 49) showed myocardial inflammation. The spontaneous course of patients with EMB-proven HHV6 DNA-associated lymphocytic myocarditis (*n* = 26) showed significant improvements in the left ventricular ejection fraction (LVEF) and clinical symptoms, respectively, in 15/26 (60%) patients, 3–12 months after disease onset. EMB mRNA expression of components of the NLRP3 inflammasome pathway and protein analysis of cardiac remodeling markers, analyzed by real-time PCR and MALDI mass spectrometry, respectively, did not differ between HHV6-positive and -negative patients. In another cohort of patients with ongoing symptoms related to lymphocytic myocarditis associated with cardiac levels of HHV6-DNA copy numbers <500 copies/µg cardiac DNA, quantified by real-time PCR, the efficacy and safety of steroid-based immunosuppression for six months was investigated. Steroid-based immunosuppression improved the LVEF (≥5%) in 8/10 patients and reduced cardiac inflammation in 7/10 patients, without an increase in cardiac HHV6 DNA levels in follow-up EMBs. Conclusion: Low HHV6 DNA levels are frequently detected in the myocardium, independent of inflammation. In patients with lymphocytic myocarditis with low levels of HHV6 DNA, the spontaneous clinical improvement is nearby 60%. In selected symptomatic patients with cardiac HHV6 DNA copy numbers less than 500 copies/µg cardiac DNA and without signs of an active systemic HHV6 infection, steroid-based therapy was found to be effective and safe. This finding needs to be further confirmed in large, randomized trials.

## 1. Introduction

Myocarditis is often induced by cardiotropic RNA viruses such as enteroviruses, but also the DNA of viruses such as parvovirus B19 (B19V) and human herpesvirus-6 (HHV6) is frequently detected in endomyocardial biopsies (EMB) of myocarditis patients [1,2]. Earlier results from the CaPACITY program (Cortisone in PArvovirus inflammatory CardIomyopaThY) showed that low levels of B19V DNA presence may not generally exclude immunosuppression interventions, which can be safe and effective under certain conditions [3]. Similar to B19V, HHV6 is highly prevalent in healthy and non-healthy individuals [4]. HHV6 has been classified into two distinct viruses, HHV6A and HHV6B, the latter being more commonly reported [5]. Both variants can infect a wide-range of human tissues and organs and preferentially replicate in host T lymphocytes, yet they depend on different host cell receptors for internalization [6,7,8]. Around 95% of adults worldwide are seropositive for either or both variants [6]. In children (<2 years of age), HHV6B has been associated with inflammatory cardiomyopathy during primary infection. Interestingly, in a child, treatment of acute HHV6 myocarditis with a left ventricular (LV) assist device and artesunate resulted in the resolution of inflammation and improvement of the clinical status and heart function [9]. Most herpesviruses maintain latency by forming circular episomes in the nucleus of the cell. In rare cases, HHV6 establishes a persistent/latent infection by integration into telomeres of chromosomes, rather than forming episomes [10,11,12]. The virus may reactivate more often in immunocompromised than in immunocompetent patients, causing neurologic manifestations and malignancies [6,13]. Otherwise, HHV6 persistence is generally considered harmless [14].

The European Society of Cardiology consensus is restrictive for the use of immunosuppressive therapy when the viral genome is present since it is not clear whether the therapy would favor viral reactivation and replication [15]. On the other hand, chronic cardiac inflammation is the most prominent predictor of poor prognosis [16], where early treatment of inflammation can be lifesaving. Within the CaPACITY program, we initiated a pilot study investigating the following: (1) Detection of HHV6 DNA in the heart of HF patients and its association with myocarditis, (2) the spontaneous course of HHV6 DNA-associated myocarditis, (3) the regulation of the NLRP3 inflammasome pathway and structural protein signatures on EMB level, and (4) the safety and effectiveness of immunosuppression in HHV6 DNA-positive patients with cardiac inflammation.

## 2. Materials and Methods

### 2.1. Patients and Study Design

**Figure 1:**
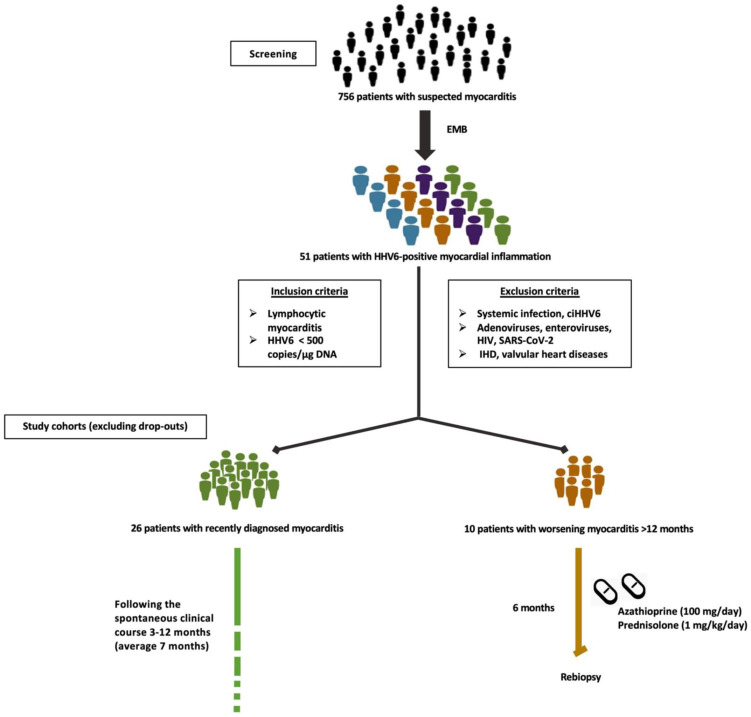
Study design. EMB, endomyocardial biopsy; HHV6, human herpesvirus 6; ciHHV6, chromosomally integrated human herpesvirus 6; HIV, human immunodeficiency virus; SARS-CoV-2, severe acute respiratory syndrome coronavirus 2; IHD, ischemic heart disease.

Patients with persistent symptoms of unexplained heart failure (HF), who do not improve despite conservative therapy, are indicated for EMB-guided diagnosis [15]. All subjects gave their informed consent for inclusion before they participated in the study. The study was conducted in accordance with the Declaration of Helsinki, and the protocol was approved by the Ethics Committee of Berlin (ethics application number EA2/140/16). At our cardiomyopathy center, 756 consecutive patients with symptoms suggestive of myocardial disease underwent catheter-guided LV-EMB procedures in the period from January 2015 until December 2020 [17,18]. EMB samples as well as blood samples were analyzed at the Cardiopathology unit of the Institute for Pathology and Neuropathology Tübingen University Hospital. The viral genome copy number was evaluated in EMB as well as in peripheral blood mononuclear cells (PBMCs), whenever possible, by a nested and quantitative real-time polymerase chain reaction (RT-PCR) as described elsewhere [19,20]. When HHV6 DNA was detected only in the nested PCR but not in the first RT-PCR, low copy numbers are reported. Only patients whose EMB revealed HHV6 DNA-associated lymphocytic myocarditis as detected by histology/immunohistology, with a copy number <500 copies/µg DNA, were selected for further investigation. Patients with systemic infection, HHV6 integrated in the genome (ciHHV6), myocardial presence of RNA/DNA of enteroviruses, adenoviruses, or patients with known human immunodeficiency virus infection or with severe acute respiratory syndrome coronavirus 2 (SARS-CoV-2) infection or other cardiac diseases (e.g., ischemic heart disease, valvular disease) were excluded. In total, 38 patients matched the inclusion and none of the exclusion criteria. Twenty-eight patients presented with recent-onset myocarditis, of which 2 dropped out after initial examination. The other 10 patients had ongoing or worsening myocarditis (>12 months). The study design is fully illustrated in Figure 1.

Recent-onset myocarditis patients (*N* = 26, left ventricular ejection fraction (LVEF) = 42 ± 13%, age: 48 ± 18 years, 16 males) were closely monitored over a spontaneous course of 3 months up to 1 year, median = 7 months. Two patients had HHV6A and 24 had HHV6B infection. None of these patients had histologically proven acute myocarditis. The severity of HF symptoms was quantified with the New York Heart Association (NYHA) classification. N-terminal pro-brain natriuretic peptide (NT-proBNP) plasma levels were analyzed via Elecsys 2010 (Roche Diagnostics GmbH, Mannheim, Germany). LVEF was measured echocardiographically using the biplane Simpson’s method. Patient characteristics are summarized in Table 1.

In another cohort of symptomatic HHV6-postive myocarditis patients, where DNA levels were quantified by real-time-PCR (*N* = 10, 8 males, LVEF = 43 ± 17%, age: 38 ± 13), a combined immunosuppression regimen, conventionally used for virus-negative myocarditis, was investigated. The patients had HHV6B DNA-associated myocarditis, ongoing or worsening symptoms (>12 months), despite standard heart failure medications, without signs of a systemic infection, and agreed to be treated with immunosuppression.

The treatment consisted of six-month 100 mg azathioprine and 1 mg/kg prednisolone once daily, and the latter was tapered off by 10 mg biweekly [21]. Following the course of immunosuppressive treatment, a follow-up biopsy was acquired from each patient to assess the efficacy and the safety of the treatment with respect to myocardial inflammation and the value of the viral copy number, respectively. The grade of inflammation is defined as grade I ≥14 leucocytes/mm^2^, ≥7 cells/mm^2^ CD3-positive T-lymphocytes and grade II ≥28 leukocytes/mm^2^, ≥14 cells/mm^2^ CD3-positive T-lymphocytes. Two patients had grade I and eight patients grade II of inflammation. The patient characteristics are summarized in Table 2.

Routine clinical follow-ups including hemodynamic examinations and transthoracic echocardiographic evaluation by independent experts were conducted in all patients. All patients received standard HF medications including renin-angiotensin-aldosterone system inhibitors, beta blockers, and according to their symptoms, diuretics and anti-anginal medications.

### 2.2. Gene Expression Analysis

RNA was isolated via the Trizol method from homogenized EMB specimens of HHV6 DNA-positive and -negative patients. Pre-amplification was performed using PreAmP MasterMix (Thermo Fisher Scientific, Waltham, MA, USA) and pooled reporter assays, as described previously [22]. Complementary DNA (cDNA) synthesis was carried out using a high-capacity reverse transcriptase kit (Thermo Fisher Scientific, Waltham, MA, USA). To quantify the gene expression level of target genes in EMB, the following Taqman reporter assays were used: NOD2 (Hs01550763_m1), NLRP3 (Hs00918082_m1), ASC (Hs01547324_gH), and IL1β (Hs00174097_m1). Expressions were normalized towards the housekeeping gene Cyclin Dependent Kinase Inhibitor 1B (Hs00153277_m1). The 2^–ΔCt^ method was used to quantify absolute gene expression.

### 2.3. Matrix-Assisted Laser Desorption Ionization-Imaging Mass Spectrometry

Matrix-assisted laser desorption ionization-imaging mass spectrometry (MALDI-IMS) was performed on 7 µm slices of EMBs from different patient groups, as described previously [23,24]. For a detailed methodology, please see the Supplementary File S1 “MALDI imaging mass spectrometry detailed methodology”.

### 2.4. Statistical Analysis

Statistical analysis of data was carried out using GraphPad Prism version 8.01. Continuous variables are expressed as mean ± SD. Paired clinical measurements were compared by paired t-test. To investigate the change of the inflammation grade over time, a generalized estimating equation (GEE) model was used to account for dependent repeated ordinal measurements.

Absolute gene expression values were compared between different groups via the Kruskal–Wallis test with Dunn’s correction for multiple comparisons. Statistical significance was assumed if a null hypothesis could be rejected at *p* < 0.05.

## 3. Results

### 3.1. HHV6 DNA Occurrence in Heart Failure and Myocarditis

**Figure 2:**
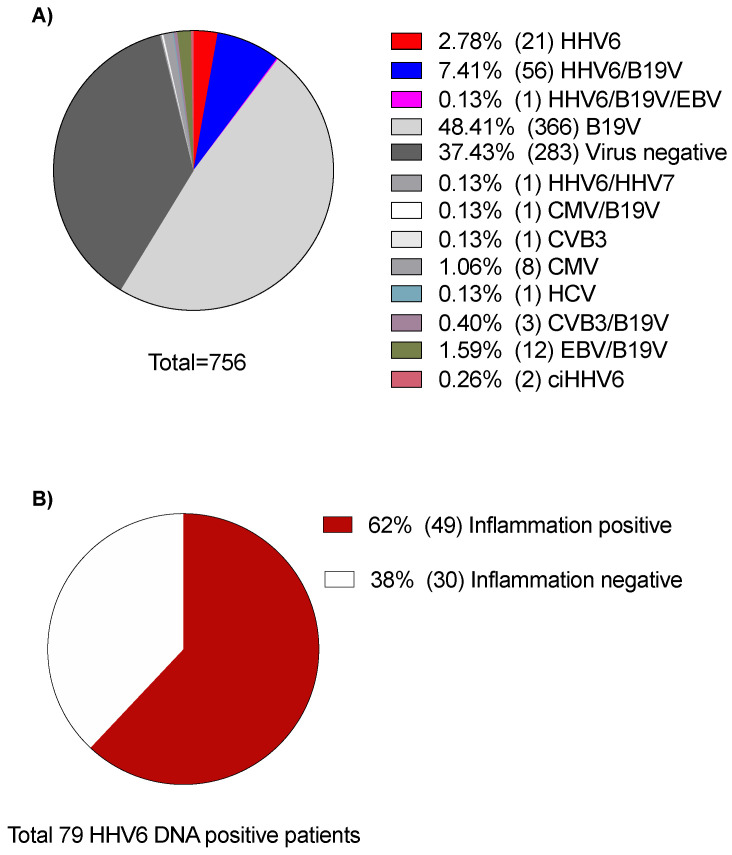
Distribution of viral genomes in endomyocardial biopsies of 756 consecutive patients clinically suspected of inflammatory cardiomyopathy or acute myocarditis. (**A**) Pie chart representing the proportions of cardiotropic virus-genomes. (**B**) Pie chart representing the occurrence of myocardial inflammation among 79 HHV6 DNA-positive patients, excluding ciHHV6. Percentages and absolute numbers were calculated based on endomyocardial biopsy findings from patients admitted to the cardiology department of Charité-Campus Virchow Clinics, at the time between January 2015 and December 2020. HHV6, human herpesvirus 6; B19V, parvovirus B19V; CVB3, coxsackievirus B3; EBV, Epstein Barr virus; HHV7, human herpesvirus 7; HCV, hepatitis C virus; CMV, cytomegalovirus; ciHHV6, chromosomally integrated HHV6.

Viral genome analysis of EMB samples revealed HHV6 DNA presence in EMBs of 79/756 patients (10.4%). Three were HHV6A positive (3.8%) and 76 were HHV6B positive (96.2%). In addition to 2/756 patients who had ciHHV6A, myocardial inflammation was present in 49/79 patients (62%), including patients with different types of myocarditis/myocardial diseases, as shown in Figure 2. Mapping of our patients according to the final clinical diagnosis revealed the presence of HHV6 DNA in low copy numbers (<500 copies/µg DNA) in 38 of 255 patients (15%) with lymphocytic myocarditis, 6 of 83 patients with dilated cardiomyopathy (DCM, 7%), 6% (1/17) of hypertrophic cardiomyopathy (HCM) patients, 4% (1/27) of cardiac amyloidosis patients, 18% (2/11) of cardiac sarcoidosis patients, 33% (1/3) of eosinophilic myocarditis patients, 25% (1/4) of restrictive cardiomyopathy (RCM) patients, and 8% (29/354) of patients whose biopsy findings showed no myocardial disease.

### 3.2. Spontaneous Course of HHV6 DNA-Associated Myocarditis

**Figure 3:**
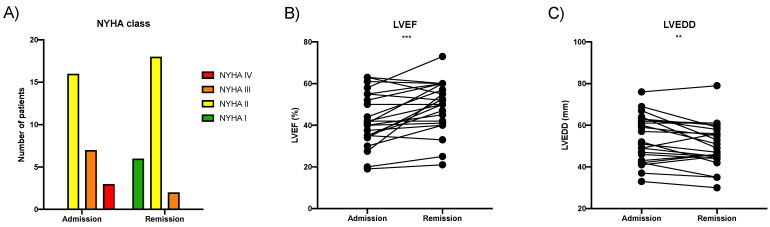
Patients with HHV6 DNA-associated myocarditis may improve spontaneously. (**A**) Columns represent the New York Heart Association (NYHA) functional class distribution of the patients at admission and after spontaneous remission. Connected dots represent the individual changes in (**B**) left ventricular ejection fraction (LVEF), (**C**) LV-end-diastolic diameter (LVEDD) between admission and spontaneous remission, *N* = 26. Statistically tested via paired *t*-test, ** *p* ≤ 0.01, *** *p* ≤ 0.001.

Many patients of the untreated cohort with lymphocytic myocarditis showed spontaneous clinical improvement, as shown in Figure 3. The New York Heart Association (NYHA) functional class dropped by one class, two classes, or remained stable in 13, 2, and 11 patients, respectively. LVEF measured via transthoracic echocardiography improved from 42.6 ± 13.4% to 48.8 ± 13.1% (mean ± SD, *p* < 0.001) where 15/26 patients improved by ≥5%. The left ventricular end-diastolic diameter reduced by 3.0 ± 5.4 mm (mean ± SD, *p* < 0.01). NT-proBNP levels were reduced from 1213 [153–2420] ng/L to 298 [66–799] ng/L (median [IQR], *n* = 19, *p* < 0.01). Baseline EMB analysis revealed mild lymphocytic infiltrates. A follow-up biopsy was not performed in these improving patients. HHV6 DNA was simultaneously detected at low copy numbers in the PBMCs of 8/26 patients.

### 3.3. Innate Immune Gene Expression in HHV6-Positive versus -Negative Endomyocardial Biopsy

**Figure 4:**
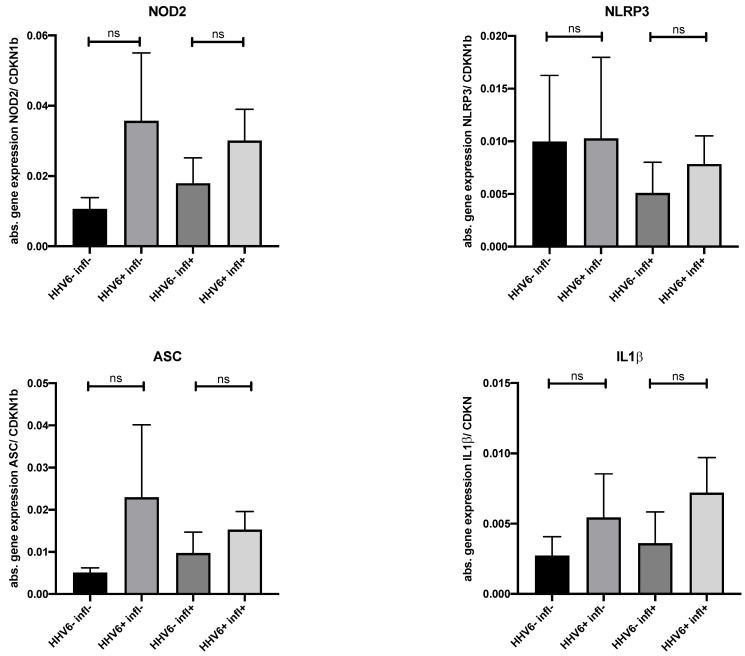
HHV6 DNA presence in the myocardium has no significant effect on the mRNA expression of NOD-like receptor pyrin domain-containing-3 inflammasome components both in presence and absence of myocardial inflammation. Bars represent the absolute gene expression values as mean ± SEM. Cyclin Dependent Kinase Inhibitor 1B (CDKN1b) was used as endogenous control. NOD2, nucleotide-binding oligomerization domain-containing protein 2; ASC, adaptor protein ASC; IL1β, interleukin 1-beta. *N* = 4–6 patients/group. ns *p* ≥ 0.05.

To investigate the impact of HHV6 DNA presence on the expression of components of the inflammasome 3 (NLRP3) pathway, gene expression analysis of EMB tissue from HHV6-positive and virus-negative patients with mild or no myocardial inflammation was carried out. There were no significant differences in the mRNA expression of the NLRP3 inflammasome components between the two groups, with and without inflammation. In addition, EMBs from patients without myocarditis or viral DNA tended to display lower mRNA expression levels of nucleotide-binding oligomerization domain-containing protein 2, adaptor protein ASC, and interleukin-1 beta compared to the other groups (*N* = 4–6 patients per group, Figure 4).

### 3.4. HHV6-Associated Myocardial Protein Signatures

**Figure 5:**
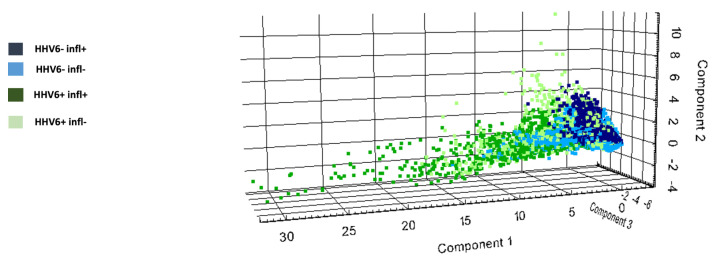
Discriminatory peptide signatures within groups of patients with clinical symptoms of myocarditis. 2D scores plot of MALDI IMS-derived spectra for components 1, 2, and 3 shows possibility of distinguishing endomyocardial biopsy peptide signature of patients with human herpesvirus 6 (HHV6) and myocarditis (dark-green cluster), myocarditis without HHV6 (dark blue), HHV6 without myocarditis (light-green cluster), and myocarditis-negative without HHV6 (pale-blue cluster).

**Figure 6:**
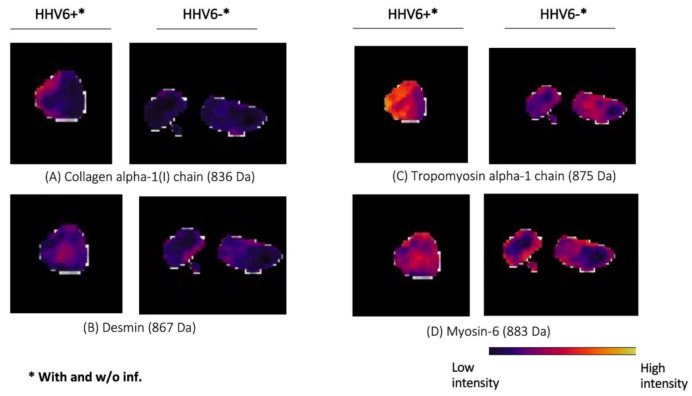
Endomyocardial biopsies of HHV6-positive and -negative patients display differential intensity distribution in selected proteins. Relative peptide expression (color bar) is shown for MALDI *m*/*z* ion peaks of (**A**) collagen alpha-1(I) chain (836 Da), (**B**) desmin (867 Da), (**C**) tropomyosin alpha-1 chain (875 Da), (**D**) Myosin-6 (883 Da) chain (833 Da), which are higher in endomyocardial biopsy tissue from HHV6-positive patients compared to the virus-negative patients. (AUC > 0.6, <0.4 *p* < 0.001).

Peptide signatures obtained from MALDI-IMS data distinguished EMB from HHV6-negative patients versus HHV6-positive patients with/without myocardial inflammation, as shown in Figure 5. Receiver operator characteristic (ROC) analysis was applied to the aligned *m*/*z* peaks from EMB of the different groups in paired comparisons. This analysis assigned 165 of the 559 *m*/*z* values (Supplementary Table S2) to peptides corresponding to proteins identified by nanoLC-MS/MS. The abundances of the different peptides (29 *m*/*z* values) from 12 proteins (CO1A1, CO6A3, DESM, HBA, H13, H4, MYH13, MYH6, PGAM2, SAMP, TPM1, TBB5) were decreased in the heart tissue of HHV6-positive patients in comparison to HHV6-negative patients, regardless of the myocardial inflammatory status. Thirteen *m*/*z* values corresponding to 13 proteins were discriminative between myocarditis patients with HHV6 and without HHV6, as shown in Figure 6. From this, the peptides of ACTA, ATPA, CO1A1, CO1A2, HSP71, H4, MYH6, PGAM2, and TPM1 were decreased and the peptides of COL6A3, G3P, HSP71, and H13 increased in HHV6-negative myocarditis patients in comparison to HHV6-positive myocarditis patients. The corresponding peptides (20 *m*/*z* values) of eight proteins (GRP78, ACTA, H13, H4, MYH6, PGAM2, SAMP, TPM1) are decreased in EMB from patients with HHV6 in contrast to patients with HHV6 and myocarditis, as shown in Supplementary Figure S1.

### 3.5. Combined Immunosuppression in HHV6 DNA-Associated Myocarditis; Pilot Study Results

**Figure 7:**
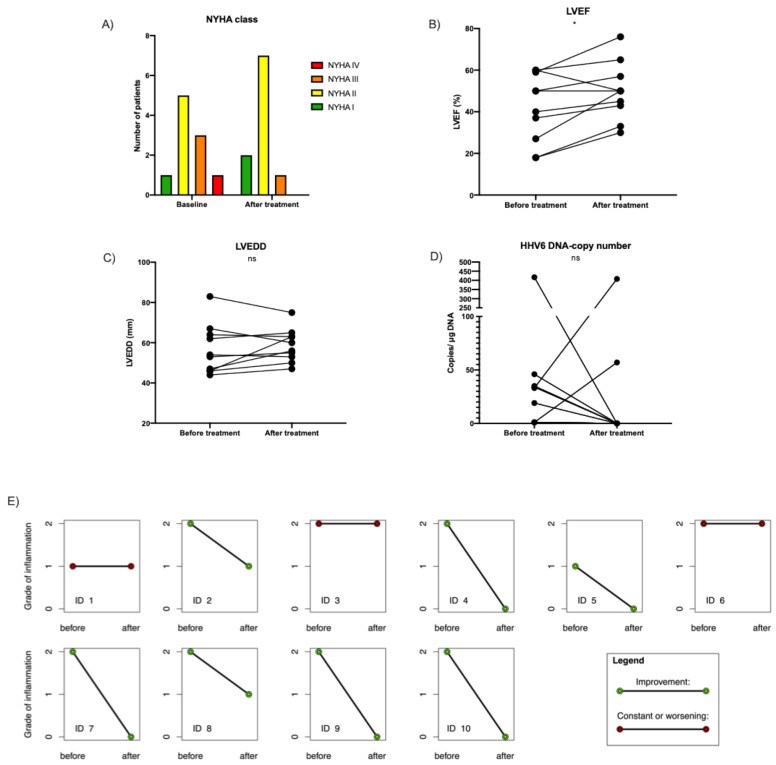
Patients with HHV6 DNA-associated myocarditis treated with steroid-based immunosuppression. (**A**) Columns represent the New York Heart Association (NYHA) functional class distribution of the patients before and after treatment. Connected dots represent the individual changes in (**B**) left ventricular ejection fraction (LVEF), (**C**) LV-end-diastolic diameter (LVEDD), (**D**) HHV6 DNA copy numbers, and (**E**) grade of inflammation (defined as grade I: ≥14 leucocytes/mm^2^, ≥7 cells/mm^2^ CD3-positive T-lymphocytes and grade II ≥28 leucocytes/mm^2^, ≥14 cells/mm^2^ CD3-positive T-lymphocytes) following six months of combined immunosuppression treatment. *N* = 10. Statistically tested via paired *t*-test, * *p* < 0.05, ns: *p* ≥ 0.05.

Following the course of treatment, the immunosuppression cohort showed an improvement in NYHA class in 4/10 patients, the complete resolution of inflammation in 5/10 patients, and a reduction of inflammation by one grade in another two patients. Analysis of change in inflammation before and after the intervention using GEE showed a significant improvement by a decrease in the ordinal inflammation grade with *p* = 0.002. The LVEF improved from 41.9 ± 16.6% to 49.9 ± 13.8% (*p* < 0.05) including 8/10 patients who improved by ≥5%, and a reduction of LVEDD by 2.1 ± 7.2 mm (*p* > 0.05). NT-proBNP levels were reduced from 623 [135–2624] ng/L to 80 [33–143] ng/L (median [IQR], *n* = 9, *p* < 0.05). Importantly, following immunosuppression, HHV6 DNA was undetectable in 8/10 patients by real-time PCR. In none of the patients did HHV6 levels increase, nor was an active HHV 6-infection induced. The treatment was safe in all patients. No drug-related adverse events, including infections, occurred. Results are represented in Figure 7. HHV6 DNA was simultaneously detected in the PBMCs of 3/10 patients at baseline and 0/10 patients after treatment.

## 4. Discussion

Although over 90% of adults are seropositive for HHV6 [25], its significance in the myocardium has not been comprehensively studied. Our study revealed that low-level HHV6 DNA, defined as detection by nested PCR, only in the second PCR, was present in EMB specimens of 10.4% of 756 patients with unexplained HF, including patients with different forms of myocarditis, genetic cardiomyopathies, storage diseases, or coronary heart disease. Similar, low levels of HHV6 could be detected in about 8% of patients without HF.

Stratification of our cohort based on clinical diagnosis shows that the cardiac HHV6 genome can be detected in a wide range of different HF forms. This agrees with the fact that persistent/latent HHV6 infections are common. In detail, in approximately 40% of patients we found that the presence of cardiac HHV6 DNA was not associated with inflammation. In most of our HHV6 DNA-positive cases, the HHV6B variant was detected, which is in line with others [26]. Only five cases showed the HHV6A variant. Independent of the virus subtype, we demonstrate that low levels of HHV6 DNA do not per se induce cardiac inflammation.

About 15% of our lymphocytic myocarditis cohort was HHV6-DNA positive, which is slightly higher compared to patients with DCM, HCM, and RCM and patients without HF. This numeric difference may reflect a patient selection bias, since the analyzed patient number without inflammatory cardiomyopathies is smaller. The clinical significance of this finding is not yet clear. On one hand, HHV6 has been linked to cardiac functional impairment [27], especially in children experiencing primary systemic active HHV6 infection where virus particles were detected in the endothelium [28]. Thus, it is well accepted that systemic HHV6 infection with high levels of HHV6 and viremia can induce acute myocarditis. On the other hand, the meaning of low levels of cardiac HHV6 DNA in lymphocytic myocarditis in unclear. In a previous study, cardiac magnetic resonance imaging findings describe predominantly macrophage-rich inflammation and myocyte damage, which was associated with the presence of B19V and HHV6 DNA in 19 cases [29]. However, proof of direct causality of these findings is still missing. To gain more insight into this issue, we analyzed the spontaneous clinical course of patients with lymphocytic myocarditis in the presence of cardiac HHV6 DNA. Initially, most of those patients showed preserved to mildly reduced LVEF, yet clinically suffered from dyspnea and exercise intolerance. About 60% of the patients exhibited spontaneous recovery in 3–6 months, which is comparable to that of uncomplicated lymphocytic myocarditis patients [30]. Interestingly, we detected HHV6 DNA in the PBMCs of 8/26 patients, which likely reflects the persistence/latency of HHV6 in blood cells. Thus, the cardiac presence of HHV6 DNA may be attributed to persistently/latently infected lymphocytes within EMBs, which may release viral DNA in the plasma and other organs following lysis [31,32]. This hypothesis is in line with the fact that, in the brain, HHV6 DNA can also persist for a long time after viremia [33]. Moreover, we conclude that the clinical improvement of the patients is most probably attributed to a spontaneous resolution of the inflammation and the presence of low-level HHV6 DNA reflects persistent/latent infection and not acute virus replication. A hypothesis-free analysis via MALDI-IMS was carried out on EMB samples to clarify whether HHV6 DNA is involved in cardiac remodeling and structural protein regulation. The analysis revealed a discriminatory peptide signature for HHV6-positive patients. However, the regulated proteins were very heterogenous and could not be related to the pathogenesis of myocarditis, allowing us to suggest that at least in patients with mild inflammation, cardiac HHV6 DNA presence does not induce, in general, a clear HHV6-specific signature associated with a typical cardiac remodeling pattern. Similarly, in a small subgroup of patients, we investigated the activation of the NLRP3 pathway, which is often involved in non-viral and viral-induced myocarditis [34,35,36,37]. However, no quantitative changes were found in EMB NLPR3 pathway gene expression patterns. Although we cannot exclude that other, more HHV6-specific inflammatory pathways were involved at other time points of the disease course, our first preliminary data suggest that the NLRP3 pathway seems not to be regulated in the presence of low-level HHV6 DNA, at least in patients with mild inflammation [34,38,39].

However, 40% of our patients still suffered from HF symptoms indicating that in some patients, cardiac inflammation may have not resolved. Untreated patients with persistent cardiac inflammation are known to have an increased risk for an unfavorable outcome [16,40]. A treatment option could be an intervention with steroid-based immunosuppression [21]. However, the ESC recommends immunosuppression only in patients without cardiac viral presence [15]. This global recommendation does not differentiate between the various (e.g., cytolytic) viruses and their infectious status and needs further evaluation for more precise restrictions.

Arguments regarding the need for better differentiation in this recommendation are the actual recommendation for the use of immunomodulating drugs including steroids or colchicine in severe COVID-19 patients [41,42], and findings of our former pilot study showing that immunosuppressive therapy can be beneficial in selected patients with ongoing lymphocytic myocarditis and B19V presence [3,21]. Considering that low-level HHV6 persistence/latency is also detected in other chronic inflammatory diseases including rheumatic diseases, where immunosuppression is usually applied without investigating the presence of viruses in the tissue, we suggest that this treatment option could be also applicable for patients with low levels of cardiac HHV6 DNA. Here, the role of immunosuppression needs to be clarified especially for those patients whose cardiac function and symptoms do not improve over time. However, such an approach is challenging knowing that on one side, HHV6 can be reactivated especially in immunocompromised patients and during infections with other viruses, such as B19V [43,44], but on the other side, inflammation per se can induce HHV6 replication in response to different cytokines [45,46]. The latter observation would even be in favor of immunosuppressive therapy. Here, our data show for the first time that the combination of prednisolone and azathioprine was safe and able to abate inflammation in 70% of carefully selected and monitored lymphocytic myocarditis patients with ongoing or worsening HF symptoms and persistent/latent HHV6 infection. Therefore, immunosuppressive interventions may not be ruled out in general [47,48].

Importantly, the copy number of HHV6 DNA did not increase after immunosuppression, which confirms the safety of the treatment in our patients. In line with this, HHV6 DNA was detected in the circulating PBMCs and the myocardium in three out of ten patients, although it cleared in PBMCs and remained in the myocardium in one out of three patients after combined immunosuppression therapy. NYHA class did not improve in 6/10 patients despite LVEF improvement and the reduction/clearance of inflammation. In those patients, a primary cardiac limitation as a cause for limited exercise capacity is unlikely, since NT-proBNP levels were also normalized or significantly reduced. However, the extended physical function limitation could be attributed to the side effect of catabolic intervention combined with physical deconditioning [49] or could belong to the consequence of a post-viral fatigue syndrome, which is also well known for chronic B19V as well as SARS-CoV-2 infection [50,51].

In conclusion, myocardial low levels of HHV6 DNA can be detected by nested PCR in different inflammatory and non-inflammatory HF forms as well as in patients without HF. There is no strong association between lymphocytic myocarditis and the presence of low levels of cardiac HHV6 DNA. Spontaneous amelioration occurs in about 60% of the cases and can be further improved by immunosuppressive therapy at least in patients with cardiac DNA levels no higher than 500 copies/µg cardiac DNA. We conclude that selected patients with ongoing myocarditis-related symptoms in the absence of systemic severe active HHV6/ciHHV6 infection, usually characterized by >1000–100,000 copies/µg DNA, may gain a similar, long-lasting, and safe benefit from immunosuppressive treatment, as do virus-negative individuals [52]. This needs to be verified in larger controlled trials.

## Figures and Tables

**Table 1:** Characteristics of the spontaneous cohort patients at admission and remission. Human herpes virus 6, HHV6; LVEDD, left ventricular end-diastolic diameter; LVEF, left ventricular ejection fraction; NYHA, New York Heart Failure Association functional class.

Patient #	Age	Sex	HHV6 Type	HHV6-DNA Copy Number	LVEF at Admission	LVEF at Remission	LVEDD at Admission	LVEDD at Remission	NYHA at Admission	NYHA at Remission
1	47	female	B	26	40	41	51	47	3	2
2	51	female	B	+	40	45	57	56	2	1
3	27	male	B	4	55	60	59	55	2	2
4	53	female	B	96	42	42	33	30	2	2
5	61	female	B	+	42	50	49	56	2	2
6	44	male	B	222	19	21	76	79	4	2
7	46	male	B	+	37.5	40	63	58	3	2
8	51	male	B	33	35	50	63	60	2	1
9	25	female	B	+	55	60	37	35	2	2
10	18	male	B	+	55	52	52	42	2	2
11	34	male	A	447	63	60	43	46	2	2
12	59	male	A	790	35	33	62	61	3	2
13	58	male	B	257	20	25	67	53	4	3
14	24	male	B	+	52	60	49	45	2	1
15	55	female	B	176	34	52.5	47	45	2	1
16	58	male	B	+	55	60	61	61	2	2
17	51	female	B	+	44	57	42	35	3	3
18	46	male	B	+	35	47	60	51	2	2
19	38	female	B	+	61	60	42	46	2	2
20	26	male	B	+	63	55	41	45	3	2
21	53	male	B	96	50	50	33	30	2	1
22	44	male	B	222	19	21	76	79	2	2
23	38	male	B	337	27.5	55	69	59	2	2
24	46	male	B	+	30	40	63	58	2	2
25	66	female	B	266	41	60	64	49	4	2
26	57	female	B	28	58	73	46	44	3	2

**Table 2:** Baseline and post-treatment characteristics of the immunosuppression treatment patients. Human herpes virus 6, HHV6; LVEDD, left ventricular end-diastolic diameter; LVEF, left ventricular ejection fraction; NYHA, New York Heart Failure Association functional class; ND, not detected.

Patient #	1	2	3	4	5	6	7	8	9	10
Age	51	29	57	33	33	38	20	48	19	31
Sex	male	female	male	male	male	male	male	female	male	male
HHV6 type	B	B	B	B	B	B	B	B	B	B
HHV6-DNA copy number before treatment	33	46	417	1	34	35	1	1	19	1
HHV6-DNA copy number after treatment	408	0	0	57	0	0	0	0	0	0
HHV6-DNA presence in PBMCs before treatment	0	0	0	+	0	+	0	0	+	0
HHV6-DNA presence in PBMCs after treatent	0	0	0	0	0	0	0	0	0	0
LVEF before treatment (%)	40	60	27	60	18	37	50	50	59	18
LVEF after treatment (%)	45	50	50	60	33	43	57	50	76	30
LVEDD before treatment (mm)	64	54	67	44	83	62	46	53	47	46
LVEDD after treatment (mm)	63	53	60	47	75	65	50	55	56	63
NYHA class before treatment	2	2	3	2	4	3	2	3	1	2
NYHA class after treatment	2	2	2	1	2	3	2	2	1	2
Grade of inflammation before treatment	I	II	II	II	I	II	II	II	II	II
Grade of inflammation after treatment	I	I	II	ND	ND	II	ND	I	ND	ND

## Data Availability

The data presented in this study are available on request from the corresponding author. The data are not publicly available due to the local patient data protection regulations.

## Supplementary Materials

The following supporting information can be downloaded at: https://www.mdpi.com/article/10.3390/v14020299/s1, Figure S1: Endomyocardial biopsies of Herpesvirus 6-positive patients with and without myocarditis display differential intensity distributions in selected proteins; File S1: MALDI imaging mass spectrometry detailed methodology.
