# Supplementary material for: The Spontaneous Course of Human Herpesvirus 6 DNA-Associated Myocarditis and the Effect of Immunosuppressive Intervention
Source: Viruses. 2022 Jan 31;14(2):299. doi: 10.3390/v14020299 (PMC8879301; doi:10.3390/v14020299)
Supplement: Supplementary file 1 [file viruses-14-00299-s001.zip › viruses-1565743-supplementary.pdf]

Supplementary material

File S1

MALDI imaging mass spectrometry detailed methodology

7 µm sections were prepared from paraffin blocks and transferred onto Indium-Tin-Oxide slides (Bruker Daltonik). Next, sections were dewaxed twice in xylene for 3 min each and passed through decreasing concentrations of ethanol (2x100%, 95% and 70%) for 1 min each. An endonuclease and trypsin solution were directly applied onto the section using an automated spraying device. Tissue incubation with the trypsin solution was performed for 3 h at 37°C in a moist chamber. Following trypsinization, the matrix solution (a-Cyano-4-hydroxycinnamic acid) was applied in ImagePrep using the manufacturer's protocol. MALDI imaging was conducted on the rapifleX® MALDI Tissuetyper® (Bruker Daltonik GmbH, Bremen, Germany) in reflector mode with the detection range of 800–3200 m/z, 500 laser shots per spot, a 1.25 GS/s sampling rate and raster width of 50µm. FlexImaging 5.1 and flexControl 3.0 software (Bruker Daltonik GmbH) coordinated the MALDI imaging run. The matrix was removed from tissue sections with 70% ethanol after MALDI imaging, and sections were stained with hematoxylin and eosin for histology. Protein identification for m/z values was performed on adjacent tissue sections using a bottom-up nano-liquid chromatography (Dionex UltiMate 3000, (Thermo Fisher Scientific, Waltham, MA, USA). electrospray ionization tandem mass spectrometry (Impact II™, Bruker Daltonic GmbH, Bremen, Germany) approach as previously described [1]. The raw spectra from the nUPLC MS/MS analysis were all converted to mascot generic files (.mgf) using the ProteoWizard software. The resulting .mgf files were then searched against a Homo sapiens protein database downloaded from the UniProt database UniProt using the Mascot search engine (version 2.4, MatrixScience). Comparison of MALDI-IMS and LC−MS/MS m/z values, requires the identification of more than one peptide (mass differences <0.5 Da). MALDI-IMS data processing for statistical analyses.

MALDI-IMS raw data were imported into the SCiLS Lab software version 2019c Pro (Bruker Daltonik GmbH) using settings preserving total ion count and without baseline removal and converted into the SCiLS base data .sbd file and .slx file. Peak finding and alignment were conducted across a data set (interval width = 0.2Da) using a standard segmentation pipeline (SciLS Lab software) in maximal interval processing mode with TIC normalization, medium noise reduction and no smoothing (Sigma: 0.75).

Discriminative MALDI-MSI m/z values from EMB tissue were identified using supervised ROC analysis. Area under the ROC curve (AUC) varies between 0 and 1, where values close to 0 and 1 indicates peptides to be discriminatory and 0.5 indicates no discriminatory value. For those peptides with an AUC >0.6 or <0.4, a univariate hypothesis test (Wilcoxon rank sum test) was used to test the statistical significance of m/z values. Peptides with p-values <0.001 were selected as candidate markers. Supervised principal component analysis (PCA) was conducted to define characteristic peptide signatures differentiating between HHV6-positive and HHV6-negative patient-groups with/without myocardial inflammation. The data were scaled for PCA in a level scaling model. Only m/z values with AUC >0.6 or <0.4 and p<0.001 were used as peak intervals for PCA using settings to create five components and use settings to use an interval width of ±0.3 Da, maximal interval processing mode, normalization to total ion count, no noise reduction. All Figures were created using the SCiLS Lab software (Bruker, Bremen, Germany).

MALDI imaging mass spectrometry supplementary figures

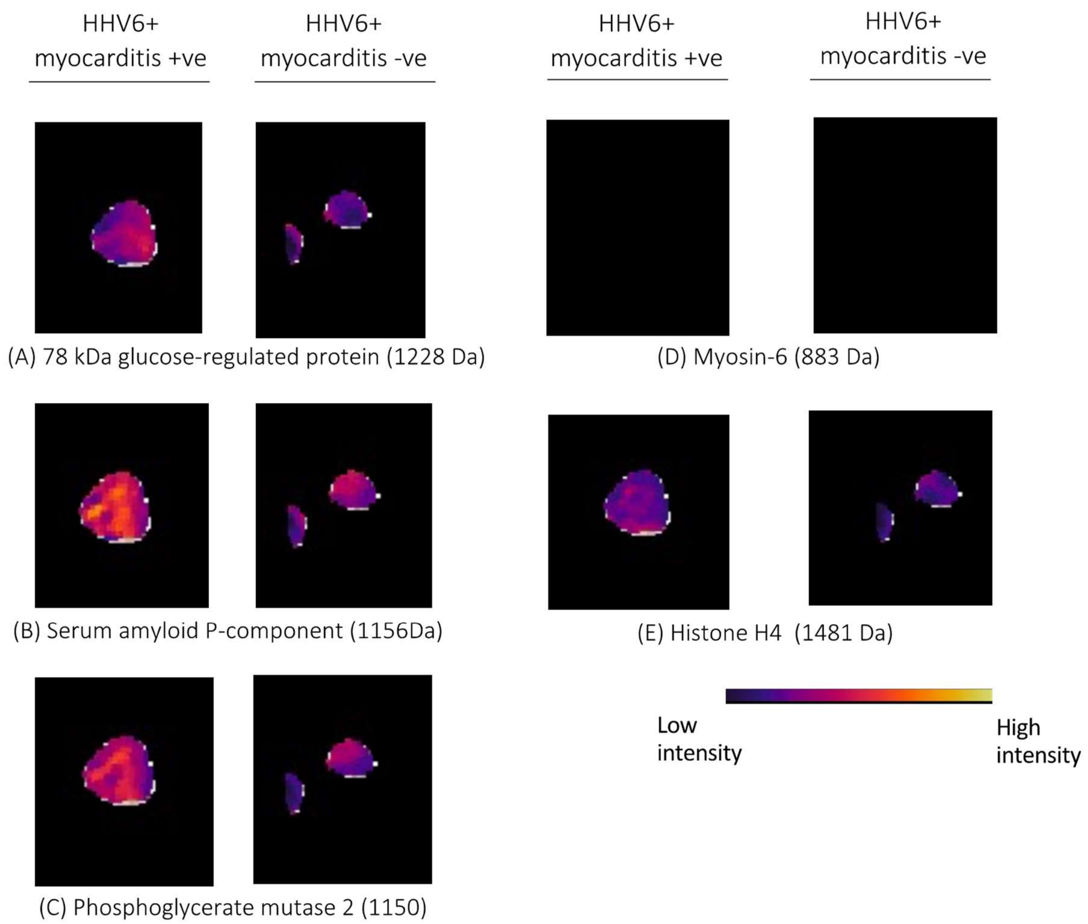


**Figure S1. Endomyocardial biopsies of Herpesvirus 6-positive patients with and without myocarditis display differential intensity distributions in selected proteins.** Relative peptide expression (color bar) is shown for MALDI m/z ion peaks of 78 kDa glucose-regulated protein (1228 Da), (B) serum amyloid P-component (1156Da), (C) phosphoglycerate mutase 2 (1150), (D) myosin-6 (883 Da), and (E) histone H4 (1481 Da), which are significantly higher in endomyocardial biopsy specimens from HHV6-positive patients (HHV6) with myocarditis compared to those without myocarditis. (AUC>0.6, <0.4 p<0.001).
